# Developmental, physiologic and phylogenetic perspectives on the expression and regulation of myosin heavy chains in mammalian skeletal muscles

**DOI:** 10.1007/s00360-023-01499-0

**Published:** 2023-06-05

**Authors:** Joseph Foon Yoong Hoh

**Affiliations:** 1 Discipline of Physiology, School of Medical Sciences, Faculty of Medicine and Health, The University of Sydney, Sydney, NSW 2006 Australia; 2 PO Box 152, Killara, NSW 2071 Australia

**Keywords:** Skeletal muscle, Myosin heavy chain, Gene expression, Myogenesis, Plasticity, Phylogeny

## Abstract

The kinetics of myosin controls the speed and power of muscle contraction. Mammalian skeletal muscles express twelve kinetically different myosin heavy chain (MyHC) genes which provides a wide range of muscle speeds to meet different functional demands. Myogenic progenitors from diverse craniofacial and somitic mesoderm specify muscle allotypes with different repertoires for MyHC expression. This review provides a brief synopsis on the historical and current views on how cell lineage, neural impulse patterns, and thyroid hormone influence MyHC gene expression in muscles of the limb allotype during development and in adult life and the molecular mechanisms thereof. During somitic myogenesis, embryonic and foetal myoblast lineages form slow and fast primary and secondary myotube ontotypes which respond differently to postnatal neural and thyroidal influences to generate fully differentiated fibre phenotypes. Fibres of a given phenotype may arise from myotubes of different ontotypes which retain their capacity to respond differently to neural and thyroidal influences during postnatal life. This gives muscles physiological plasticity to adapt to fluctuations in thyroid hormone levels and patterns of use. The kinetics of MyHC isoforms vary inversely with animal body mass. Fast 2b fibres are specifically absent in muscles involved in elastic energy saving in hopping marsupials and generally absent in large eutherian mammals. Changes in MyHC expression are viewed in the context of the physiology of the whole animal. The roles of myoblast lineage and thyroid hormone in regulating MyHC gene expression are phylogenetically the most ancient while that of neural impulse patterns the most recent.

## Introduction

### Molecular physiology of myosin

Myosin is the motor protein in muscle cells which generates force and movement of muscle fibres by hydrolysing ATP. Structurally, it is a hexamer consisting of a pair of heavy chains (MyHC) and two pairs of light chains (MLC1 and MLC2) which together form a two-headed molecule with an elongated rod-like tail. The C-terminal halves of the heavy chains are α-helical and wrap around each other to form the tail, while the N-terminal halves form the globular heads, each of which contains an ATPase site and an actin binding site. Each head is attached to the rod by a lever arm domain to which the essential light chain (MLC1) and the regulatory light chain (MLC2) are attached. In striated muscles, myosin molecules assemble into thick filaments with the heads on their surface. During muscle contraction, myosin heads act as cross-bridges that cyclically interact with actin molecules of the thin filaments, hydrolysing ATP and generating force and movement. During the chemo-mechanical coupling, small conformational change in the catalytic domain of the head gets amplified by the lever arm (Squire 2019). This cyclic activity generates the relative force and motion between the thick and thin filaments which underlie the sliding filament theory of muscle contraction (Huxley 1957).

Myosin controls the kinetics of energy transduction from ATP, and through it, the kinetic properties of muscles. The speed of contraction of a muscle is generally proportional to the ATPase activity of its myosin (Barany 1967). As the maximal stress (force/cross-sectional area) of muscles of different speeds is about the same, muscle power (force x velocity) is also a function of myosin ATPase activity. High muscle speed and power may confer an evolutionary advantage to an animal, such as escaping from predators or chasing prey. However, skeletal muscles constitute 40–45% of body mass in all mammals (Schmidt-Nielsen 1984) and, in an active animal, are the greatest consumers of energy in the body, the bulk of which is spent in cross-bridge cycling. The benefit of locomotory muscle of high speed is balanced by the high energy cost and the need for high caloric intake. Muscles of low speed are economical for producing sustained tension, the energy cost for tension maintenance is related to speed and myosin ATPase activity. The functional demands on skeletal muscle fibres in different parts of the body are highly varied and complex, ranging from providing ripple free steady tension for eye fixation, economic postural maintenance, powerful jaw closure, fast locomotion, and high-speed saccadic eye movements. To meet these functional demands, eleven different striated muscle MyHC genes exist in the mammalian genome (Desjardins et al. 2002). These MyHCs associate with various types of MLCs to form myosin isoforms with a wide range of kinetic characteristics. In addition to these striated muscle MyHCs, a member of the non-muscle MyHCs, nmMyH 2B (MYH10) is uniquely expressed as a thick filament motor protein in extraocular muscle (EOM) (Moncman and Andrade 2010), but is only expressed in the Z-lines of limb muscles and in the intercalated discs and Z-lines of cardiac myocytes (Takeda et al. 2000).

#### Historical introduction to research on muscle kinetics and myosin isoforms

Prior to 1960, it was known that all mammalian limb muscles were slow at birth, the differentiation into the fast and slow types occurred during the first few weeks after birth (see references in Buller et al. 1960a). Using the time course of isometric contractions as a measure of speed, Buller et al. showed that the differentiation of fast hindlimb muscles in the cat is virtually unaffected by spinal cord transection or surgical isolation of the lumbosacral spinal cord from all incoming impulses, whereas the developing slow muscle increased in speed relative to adult slow muscle (Buller et al. 1960a). This implies that the differentiation of slow muscle is subject to an influence exerted by motoneurons. These authors then surgically crossed the nerves supplying fast and slow muscles in adult cats and showed that their isometric contraction times were reversed, fast muscle became slow while slow muscle became fast contracting, indicating that the nerve supply has a profound influence on contractile properties of skeletal muscles (Buller et al. 1960b). These classical studies strongly stimulated research into the cellular and molecular bases for differences between fast and slow muscles. It led to works showing that the maximal speed of contraction of a muscle (Vmax) is proportional to the ATPase activity of its myosin (Barany 1967), and that cross-innervation of fast and slow muscles led to the reversal of their Vmax (Close 1965) and their myosin ATPase activity (Barany and Close 1971). The focus on myosin as the basis for muscle kinetics led to the characterization of fast-red (2a), fast-white (2b) and slow myosin isoforms in fast and slow muscles (Gauthier and Lowey 1979), their transformation after nerve cross-union (Hoh 1975), the discovery of foetal/neonatal (Hoh and Yeoh 1979), embryonic (Whalen et al. 1981), α-cardiac and β-cardiac myosins (Hoh et al. 1978, 1979), slow-tonic myosin (Pierobon-Bormioli et al. 1979), extraocular (EO) myosin (Sartore et al. 1987), 2X myosin (Bar and Pette 1988; Schiaffino et al. 1989), and the demonstration that β-cardiac myosin is identical to the slow skeletal myosin (Lompre et al. 1984), referred to hereafter as β-slow myosin.

#### Myosin heavy chain genes expressed in mammalian skeletal muscle fibres

Twelve MyHC genes known to be expressed in mammalian striated muscles, namely, the eleven striated muscle MyHC genes and the non-muscle MyHC IIB (nmMyH IIB) (MYH10) which is only expressed in extraocular muscle (Moncman and Andrade 2010). The eleven striated muscle MyHC genes include the six skeletal MyHCs which are clustered in tandem in the mammalian genome (Weiss et al. 1999) in the following order: embryonic (MYH3), fast 2A (MYH2), 2X (MYH1), 2B (MYH4), neonatal/foetal (MYH8) and EO (MYH13). There are two cardiac isoforms, α-cardiac (MYH6) and β-slow (MYH7), the genes for which are arranged in tandem in the genome (Saez et al. 1987; McNally et al. 1989), two EOM-specific isoforms, the slow-tonic (MYH14/7b), slow B (MYH15) (Rossi et al. 2010), and one highly jaw-specific and most ancient masticatory MyHC (MYH16) (Qin et al. 2002).

#### Kinetic properties of striated muscle myosin isoforms

The kinetic properties of muscle fibres and myosin isoforms can be compared using several different methods. The force–velocity relation of a fibre characterizes its speed and power as a function of load. Vmax can be obtained by extrapolating the force–velocity data of the muscle to zero load (Close 1964) or by using the slack test (Edman 1979; Moss et al. 1982). Its value is proportional to the actin-activated ATPase activity of its myosin (Barany 1967) and is correlated with the rate of detachment of cross-bridges from actin during the cross-bridge cycle, which is determined by the rate of ADP release from the cross-bridge (Siemankowski et al. 1985; Weiss et al. 2001). The kinetics of stretch-induced delayed force increase (stretch activation) is also indicative of fibre speed (Galler 1994; Andruchov et al. 2004). Fibre kinetics can also be measured in the frequency domain by dynamic stiffness analysis (Rossmanith et al. 1986}, which yields a stiffness minimum frequency, f_min_, that is correlated with the rate of cross-bridge cycling. The kinetics of purified myosin molecules can be assessed by an in vitro motility assay which measures the speed at which myosin molecules immobilized on a nitrocellulose film move fluorescently labelled actin filaments (Pellegrino et al. 2003; Resnicow et al. 2010).

As will be discussed later, the kinetic properties of myosin isoforms are generally inversely related to the animal’s body mass, but the ranking order of the kinetics of various isoforms is approximately invariant within species. Kinetic analyses on EO muscle (EOM) (Close and Luff 1974; Luff 1981; Asmussen et al. 1994) and single EOM fibres (Li et al. 2000; McLoon et al. 2011) have established that EO MyHC is the fastest isoform. Analysis of recombinant human EO myosin ATPase reveals features of fast kinetics, including the weak affinity of ADP for actin-EO myosin complex, suggesting an accelerated cross-bridge detachment rate (Bloemink et al. 2013).

Of the isoforms found in limb and trunk muscles, 2B, 2X, 2A and β-slow myosins are in descending order of speed (Hilber et al. 1999; Reggiani et al. 2000; Weiss et al. 2001). The α-cardiac myosin is two- to threefold faster than β-slow myosin (Rossmanith et al. 1986; VanBuren et al. 1995), placing its kinetic properties close to that of 2A myosin, consistent with the Vmax of α-cardiac myosin-containing rabbit skeletal muscle fibres, which lies between the Vmax of fibres expressing β-slow and 2A myosin (Sciote and Kentish 1996). The kinetics of β-slow myosin is more sensitive to temperature than that of α-cardiac myosin (Yazaki and Raben 1975; Rossmanith et al. 1986) and limb fast myosins (Li et al. 2000; Hoh et al. 2007b). This implies that β-slow myosin has a higher activation energy, reflecting a slower ADP dissociation rate from actin-myosin complex, a more stable attached cross-bridge and increased time that β-slow myosin is bound to actin (Bloemink et al. 2007). These characteristics underlie the low tension cost of slow skeletal fibres (Bottinelli et al. 1994) and hearts (Rossmanith et al. 1995) expressing β-slow MyHC.

The velocities of shortening of new-born fast and slow rat limb muscles are virtually the same as that of adult slow muscle (Close 1964). Since these muscles express predominantly foetal/neonatal myosin (Hoh and Yeoh 1979; Whalen et al. 1981; Agbulut et al. 2003), Vmax of rat neonatal myosin is thus close to that of β-slow myosin. However, model analysis of human neonatal myosin suggests that it shares some kinetic characteristics with fast myosins (Johnson et al. 2019). The kinetics of embryonic and slow B myosins have not been specifically determined but are likely to be as slow or slower than β-slow myosin (Schiaffino et al. 2015). Slow B MyHC, an orthologue of amphibian ventricular MyHC, is uniquely expressed in rat orbital EOM fibres (Rossi et al. 2010). The very slow contractions of multiply-innervated EOM fibres of the rabbit (Matyushkin 1964) and cat (Bach-y-Rita and Ito 1966; Lennerstrand 1974; Nelson et al. 1986) can be attributed to the presence of slow-tonic MyHC, which is closely related to the MyHC in slow-tonic fibres in birds and amphibians (Pierobon-Bormioli et al. 1979, 1980), which produce very slow contractures (Close and Hoh 1968a; Morgan and Proske 1984).

Cat masticatory fibres which express masticatory myosin have a very short contraction time (Taylor et al. 1973; Hoh and Hughes 1988) and masticatory myosin has a high ATPase (Rowlerson et al. 1981). These fibres were thought to be superfast contracting, but both dynamic stiffness analysis (Hoh et al. 2007b) and unloaded velocity measurements (Toniolo et al. 2008) indicated that masticatory fibres are comparable to limb fast fibres in speed of contraction. The rapid development of force may be attributed to the high Ca^2+^ sensitivity (Kato et al. 1985) and the movement of masticatory myosin heads away from the thick filament revealed by X-ray diffraction studies (Yamaguchi et al. 2010), a feature also seen during post-tetanic potentiation of limb fast muscle (see later). Masticatory fibres develop substantially more force per cross-sectional area compared with fast fibres (Kato et al. 1985; Toniolo et al. 2008), this being associated with a higher tension-cost (Saeki et al. 1987). Masticatory fibres are therefore more powerful than limb fast fibres. This attribute together with the higher rate of force development are highly appropriate for a carnivorous lifestyle.

#### Modulation of myosin kinetics by myosin light chains and MyBP-C

The kinetics of a myosin isoform is determined principally by its MyHCs. The MLCs modulate myosin kinetics, MLC1 by its N-terminus interacting with the C-terminus of actin during the cross-bridge cycle (Morano 1999) and MLC2 through its effect on thick filament structure upon phosphorylation. The essential MLC of fast myosins exits as two isoforms (MLC1f, and MLC3f) (Hoh and Yeoh 1979) which are spliced variants of the MLC1f/MLC3f gene (Barton and Buckingham 1985). These isoforms share a C-terminal sequence, but differ in the N-terminal region, MLC1f having 38 amino acid residues more than MLC3f. The functional significance of MLC1 heterogeneity is apparently the modulation of cross-bridge kinetics of fast MyHCs. Fast myosin with MLC3f moves actin filaments faster in motility assays than myosin with MLC1f (Lowey et al. 1993) and the Vmax of rabbit fast fibres is proportional to the content of MLC3f (Greaser et al. 1988). The MLC1f isoform with the large N-terminal domain has a greater affinity for actin (Stepkowski 1995), thereby retarding cross-bridge detachment rate during the cross-bridge cycle. MLC1f and MLC3f have independent transcription initiation sites (Barton and Buckingham 1985), enabling their differential regulation. Chronic stimulation of rat fast muscle enhances the ratio of MLC1f/MLC3f (Bar et al. 1989), and this is expected to reduce the speed of fast fibres, whereas hyperthyroidism enhances the expression of MLC3f (Kirschbaum et al. 1990) and would increase contraction speed.

The MLCs of embryonic (Whalen et al. 1978), atrial (Hoh et al. 1978) and masticatory (Rowlerson et al. 1981) myosins were initially thought to be distinct entities. However, later work revealed that MLC1 of masticatory myosin is identical to that of the embryonic (MLC1_E_) as well as that of the atrial (MLC1_A_) myosin (Reiser et al. 2010), and has been designated as MLC1_E/A_. The N-terminal of MLC1_E/A_ has fewer charged amino acid residues compared with ventricular MLC1 and has a weaker interaction with actin (Morano et al. 1996). The cross-bridge cycling rate of β-slow MyHC associated with slow/ventricular MLC1 is dramatically increased when this MLC1 is replaced by MLC1_E/A_ in cat jaw-slow fibres (Hoh et al. 2007b). Jaw-specific slow fibres in jaw-closing muscles hold the jaw up against gravity. Further, the temperature sensitivity of f_min_ is reduced, indicating a reduced activation energy for the cross-bridge cycling, which reflects a reduced stability of the attached cross-bridge relative to that of limb-slow fibres (Hoh et al. 2007b). The functional significance of the accelerated cross-bridge cycling rate and reduced stability of attached cross-bridge is that these features would facilitate rapid jaw opening before a powerful bite of the carnivore. In the atrium of the heart, α-cardiac MyHC is associated with MLC1_E/A_ whereas in the ventricle, α-cardiac MyHC is associated with ventricular MLC1. The MLC1_E/A_ increases the speed of atrial contraction and relaxation relative to that of the ventricle with the same α-cardiac MyHC (Morano et al. 1996), thereby ensuring more time for the ejection phase of the cardiac cycle. Some 2a fibres in rat and rabbit have both fast and slow isoforms of MCL1. The kinetic properties of these 2a fibres with fast and slow MLC1 isoforms are 20% slower than 2a fibres with purely fast MLC1 (Andruchov et al. 2006). Kinetic analysis of chimeric fast and slow myosins with different MLC variants also confirmed the role of MLC1 in modulating the kinetics of myosin (Nayak et al. 2020).

MLC2 modulates muscle contraction by a mechanism that involves its phosphorylation. The myosin heads in resting muscle fibres are generally in an ordered helical array bound to the core of the thick filament in which the two heads of each myosin interact closely with one another to form an evolutionarily conserved interacting head motif (Alamo et al. 2008). This creates a super-relaxed state (SRX) of myosin with very low Mg^2+^ ATPase activity (Stewart et al. 2010). The MLC2 in fast fibres is phosphorylated during a tetanus by Ca^2+^-calmodulin activated myosin light chain kinase (Stull et al. 1980, 2011). X-ray diffraction analysis revealed that myosin heads with phosphorylated MLC2 are shifted away from thick filaments (Yamaguchi et al. 2016) and are thus closer to thin filaments. This enhances myofibrillar Ca^2+^ sensitivity and accelerates cross-bridge attachment rate following activation (Metzger et al. 1989; Sweeney and Stull 1990), generating post-tetanic potentiation of twitch contractions with accelerated rate of rise of tension development (Close and Hoh 1968b; Sweeney et al. 1993) but without influencing Vmax (Persechini et al. 1985). Phosphorylation of MLC2 also occurs in ventricular muscle by protein kinase C (Venema et al. 1993) following α-adrenergic (Terzic et al. 1992) and endothelin (Rossmanith et al. 1997) stimulation with similar enhancement of Ca^2+^ sensitivity and twitch force enhancement. Recently, an interacting head motif of even lower ATPase activity has been described in muscles of invertebrates that can stay motionless for long periods of time (Craig and Padron 2022). The existence of this hyper-relaxed state of myosin has not been reported in mammalian skeletal muscle but is worth looking for in muscles of hibernating animals.

The release of myosin heads from SRX following phosphorylation of MLC2 has an impact on resting metabolism of the animal. Measurement of single nucleotide turnover rate of relaxed, skinned rabbit psoas muscle fibres revealed two lifetimes for nucleotide release, a fast component similar in kinetics to that of purified myosin, and a slow component that is more than 10 times slower. The slow component is attributable to the helically ordered cross-bridges in thick filaments in the SRX state while the fast component is due to cross-bridges released from the thick filament by the phosphorylation of MLC2 (Stewart et al. 2010). Stewart and co-workers suggest that myosin Mg^2+^ ATPase activity in resting muscle may play an important part in mammalian thermogenesis. As muscles make up about 40–45% of body mass, even a low level of Mg^2+^ ATPase activity in resting muscle would have a significant impact on the basal metabolic rate of the resting animal. The increase in Mg^2+^ ATPase activity of myosin isoforms in animals of decreasing body mass (see later) and the possible enhancement of the basal level of phosphorylation of MLC2 in small animals could significantly contribute to their higher basal metabolic rate.

Myosin binding protein-C (MyBP-C) also has an influence of myosin kinetics. This myofibrillar protein exists in four known isoforms: fast skeletal (fMyBP-C), slow skeletal, masticatory (m-MyBP-C) and cardiac (cMyBP-C). Much of the work on the function of MyBP-C (reviewed in Barefield and Sandayappan, 2010 and Harris 2021) has been done on cMyBP-C because mutations in cMyBP-C in humans can cause hypertrophic cardiomyopathy. MyBP-C is an elongated molecule made up of a series of Ig-like and fibronectin-like domains. The C-terminal of MyBP-C binds to the myosin tail in the thick filament while the N-terminal binds to the myosin head when cMyBP-C is not phosphorylated but to the thin filament after phosphorylation. Binding to the myosin head stabilizes the SRX (McNamara 2019). Phosphorylation of the N-terminal region of cMyBP-C destabilizes the SRX, releasing myosin heads from the thick filament and facilitates the binding of the N-terminals of cMyBP-C to actin filaments, enhancing Ca^2+^ sensitivity, accelerating cross-bridge cycling kinetics and enhancing force development (Stelzer et al. 2007; Tong et al. 2008; Moss et al. 2015). The role of skeletal isoforms of MyBP-C is less well known, but a recent study indicated that in the absence of the N-terminal of fMyBP-C, peak tetanic force and isotonic speed of contraction are significantly reduced (Song et al. 2021). The binding of fMyBP-C to actin in intact skeletal fibres has been demonstrated by electron tomography (Luther et al 2011). An immunochemically unique isoform of skeletal MyBP-C, m-MyBP-C, has been identified in masticatory fibres of the cat (Kang et al. 2010). The N-terminal of this isoform may have a weak interaction with masticatory myosin, thereby reducing SRX to allow cross-bridges to swing out towards thin filament as shown by X-ray diffraction analysis (Yamaguchi et al. 2010). Much future work needs to be done on skeletal isoforms of MyBP-C to clarify their different roles in skeletal muscle contraction.

Since the classical work of Buller et al. on the plasticity of limb muscle fibre types, many cellular and molecular mechanisms have been identified that influence the expression of MyHCs in somitic and craniofacial muscle fibres (Bassel-Duby and Olson 2006; Schiaffino and Reggiani 2011). This review will examine molecular mechanisms of MyHC gene expression of limb muscles in mammals in the context of MyHC gene expression in other parts of the body, focussing on the roles of cell lineage, neural activity, and thyroid hormone during development and in adult life. How body size influences MyHC kinetics and isoforms expressed in these muscles and how these muscles adapt to conserve energy during locomotion as body size, basal metabolic rate, and pattern of use change during phylogeny. Changes in MyHC expression are viewed in the context of the physiology of the whole animal. The evolutionary origin of the mechanisms regulating MyHC expression during phylogeny will also be briefly examined.

### Developmental origins of skeletal muscle fibres and the notion of muscle allotype

Myogenic cells forming muscles in different regions of the body arise from myogenic progenitor cells in different parts of the paraxial mesoderm of the early embryo and are committed to different lineages by diverse regulatory factors (Sambasivan et al. 2009). Limb and trunk muscles develop from cells in the segmented somites on each side of the developing spinal cord. EOMs develop from cells that originate from the prechordal plate (Wachtler et al. 1984; Couly et al. 1992). Other craniofacial muscles originate from unsegmented cranial paraxial mesoderm and the most rostral occipital somites (Noden and Francis-West 2006). The progenitor myoblasts from these regions migrate into the branchial arches. Jaw muscle cells are derived from the first branchial arch, intrinsic laryngeal muscle from the fourth arch and sixth arch (Sperber 1989). Expression of the pituitary homeobox gene 2 (Pitx2) commits progenitors from the prechordal plate to the EOM myoblast lineage, expression of T-box gene 1 (Tbx1) to branchial myoblast lineage and of Pitx3 gene to trunk and limb myoblast lineage (Sambasivan et al. 2009, 2011). Progenitor myoblasts in the branchial arches and somites are further specified by different transcription factors into various lineages and ultimately committed to myogenesis by the four myogenic regulatory factors: Myf5, Mrf4 and MyoD committing progenitor myoblasts to the myogenic fate, and myogenin, the terminal differentiation factor that initiates the differentiation of myoblasts into muscle fibres (Liu et al. 2010; Buckingham and Rigby 2014).

Across mammalian species, adult muscles of the craniofacial and somitic origins have very different repertoires for MyHC gene expression, the specific isoform expressed in a muscle fibre of a given animal is determined by functional demand of the structure the muscle moves. The repertoire of limb and trunk muscles include β-slow, 2A, 2X and 2B MyHCs. Laryngeal muscles are also able to express these isoforms, with the profile of MyHCs shifted towards faster isoforms in small animals, which also include the fastest EO MyHC, needed to enable laryngeal muscles to cope with the higher respiratory rate of smaller animals (Hoh 2005). EOMs express all striated muscle MyHC isoforms except the masticatory MyHC, but also uniquely include nmMyH 2B, a non-muscle isoform (Hoh 2021), the extremely wide range of kinetic properties enables EOMs to execute very fast saccades and provide ripple-free eye fixation. The jaw-closer muscles of different mammalian taxa express different MyHC isoforms to match the functional requirements of their diet: fast and β-slow MyHCs in rodents, β-slow MyHC in ruminants, α-cardiac MyHC in browsing macropods and the high force and highly jaw-specific masticatory MyHC in carnivores (Hoh 2002).

As muscles in these craniofacial and somitic regions express different MyHCs and other myofibrillar proteins, the question arises as to whether these properties are intrinsic to the lineages of myoblasts or are induced by some extrinsic factor such as differences in innervation. This was resolved by transplantation of cat temporalis muscle, which normally expresses the jaw specific masticatory MyHC, into limb muscle beds to be innervated by nerves to fast or slow limb muscles. While it is well known that regenerating limb muscles express β-slow or limb fast MyHCs, regenerated temporalis muscles innervated by limb fast muscle nerve expressed masticatory MyHC but not limb fast MyHCs, while regenerates innervated by limb slow muscle nerve expressed β-slow MyHC (Hoh and Hughes 1988). Regenerates expressing masticatory MyHC also expressed jaw-specific isoforms of myosin binding protein-C (MyBP-C), m-MyBP-C and tropomyosin (m-Tm) while those expressing β-slow MyHC had immunohistochemical characteristic of jaw-slow fibres (Kang et al. 2010) due to the association of masticatory MLCs rather than slow MLCs with β-slow MyHC (Hoh et al. 2007b). Myotubes derived from tissue cultures of satellite cells from cat temporalis muscle also expressed masticatory MyHC, m-MyBP-C and m-Tm (Kang et al. 2010). These results indicate that the capacity to express masticatory MyHC and other jaw-specific myofibrillar proteins is intrinsic to the temporalis myoblast lineage. The term allotype has been used to classify muscles from different body regions with different repertoires for expressing MyHCs and other myofibrillar proteins.

The notion that myogenic cells of different muscle allotypes would express their unique MyHC isoforms during myogenesis has also been tested on EOMs. Initial experiments on cultured EOM satellite cells, as well as the incorporation of EOM satellite cells into limb fast muscle fail to induce the expression of EOM-specific EO and slow-tonic MyHCs (Sambasivan et al. 2009). With a repertoire of 11 MyHCs, expression of MyHCs in regenerating EOM is understandably far more complex than that of cat jaw muscle. Regenerating rabbit EOMs innervated by limb fast muscle nerve also do not express EO MyHC (Hoh 2021). However, EOM regenerates innervated by the oculomotor nerve do express EO MyHC (Hoh 2021). The oculomotor nerve can deliver very high frequency impulses of around 700 Hz during saccades, a characteristic which may be necessary for EO MyHC expression. This frequency characteristic is absent from limb muscle nerves. Future research to test whether very high frequency impulses are specifically required for inducing EO MyHC expression in EOM transplants and cell cultures would be of great interest.

#### Fibre types and their myosins in limb and trunk muscles

Mammalian limb and trunk muscles subserve locomotion and maintenance of posture as well as skilful movements in higher mammals. Fibre types in these muscles and their plasticity have been extensively studied and reviewed (Pette and Staron 1997; Schiaffino and Reggiani 2011). These fibre types can be classified into two broad phenotypes, fast and slow. The slow fibres express β-slow MyHC. The fast fibres can be further divided phenotypically into 3 subtypes called 2a, 2x and 2b, which respectively express 2A, 2X and 2B MyHC. These four fibres provide a wide range of muscle speed, power, and endurance (Bottinelli et al. 1991). Slow myosin has a low ATPase activity and produces tension economically. Slow fibres are endowed with a high mitochondrial content, oxidative enzymes, and a rich blood supply, thus enabling them to generate a steady supply of ATP, and so have high fatigue resistance. The expression of β-slow MyHC and enzymes involved in oxidative energy metabolism is coordinated by a nuclear receptor/microRNA circuitry (Gan et al. 2013). Appropriately, slow muscles are used in postural maintenance and slow, sustained movements. The 2b fibres have high levels of glycogen and glycolytic enzymes, and can generate rapid bursts of ATP anaerobically, appropriate for short spurts of high speed and power, but they lack endurance. The 2a and 2x fibres have kinetic and metabolic characteristics between these extremes. Having fibres with different functional characteristics enables mammals to use their muscles efficiently for different types of functions.

Individual locomotory muscles in both eutherian (Lucas et al. 2000) and marsupial (Zhong et al. 2001) mammals are composed of some or all these four fibre types in different proportions. However, fibre type expressed is influenced by body size: 2b fibres are absent in large animals including humans, and slow fibres are absent in very small animals (see later). Slow muscles such as soleus and vastus intermedius are found deep in the limb close to the bone and contain slow and 2a fibres. Fast muscles such as tibialis anterior and gastrocnemius, are more superficially located and have a deep, ‘red’ region with predominantly slow and 2a fibres and a ‘white’ superficial region consisting only of fast, predominantly 2b and 2x fibres.

#### Regulation of limb and truck muscle fibre types

Since the classical works on the effects of nerve cross-union on fast and slow muscles referred to above, considerable work has shown that limb muscle fibres are highly plastic, one type of fibre can potentially be converted into another type by many factors, but principally by impulse patterns of innervating motoneurons, changes in impulse patterns associated with the pattern of use and changes in thyroid and other hormone levels (Pette and Staron 2000; Bassel-Duby and Olson 2006; Schiaffino and Reggiani 2011; Soukup and Smerdu 2015). Hennig and Lomo showed that neural impulse pattern to rat slow soleus muscle is characterized by a sustained activity at low frequencies, whereas that to the fast extensor digitorum longus (EDL) muscle is characterized by high frequency and has 2 subtypes, one of low abundance in number of impulses per day, consisting of very infrequent short bursts of high frequencies, the other of high abundance, with long bursts of high frequencies (Hennig and Lomo 1985).

The effects of different impulse patterns on MyHC expression of fast and slow muscles have been extensively investigated. Denervated EDL subject to chronic low frequency stimulation (CLFS) that simulates the pattern of nerve impulses delivered by the nerve to slow muscle rapidly induces a 2B→2X→2A MyHC transition (Termin et al. 1989), leading to the complete loss of 2B MyHC and accumulation of 2X/2A MyHCs after 2 months (Ausoni et al. 1990), while a substantial 2A→β-slow transition takes place after 4 months (Windisch et al. 1998). High frequency high-amount impulse stimulation of the denervated EDL drastically suppresses 2B MyHC expression with replacement by 2A/2X MyHCs, while high frequency low-amount stimulation has a milder effect and preserves nearly normal 2B expression in many of the fast fibres (Ausoni et al. 1990). However, in the denervated soleus muscle, high frequency low-amount stimulation induces the expression of 2A/2X but not 2B MyHC expression, while high frequency high amount stimulation induces predominantly 2A MyHC (Ausoni et al. 1990). These results show that the response to a given pattern of stimulation is muscle specific.

The conversion of a fast muscle into a slow one following cross-innervation by a slow nerve is due to CLFS delivered by the slow nerve (Pette and Vrbova 1992) which induces an incomplete sequential transition 2B→2X→2A→β slow MyHC transition (Mira et al. 1992), some fast fibres failing to express β-slow MyHC. The conversion of slow muscle into a fast one after cross-innervation by a fast nerve is thought to be due to the replacement of CLFS with high frequency impulse patterns (Gorza et al. 1988). Deprivation of CLFS in the background of the euthyroid state plays a dominant role, since reducing neuromuscular activity or depriving the neural input into limb muscles by cordotomy (Hoh et al. 1980), hindlimb suspension (Hoh and Chow 1983; Diffee et al. 1991a), spaceflight (Adams et al. 2000; Shenkman 2016) and spinal cord isolation (Huey et al. 2001) leads to inhibition of β-slow MyHC expression in slow muscles and its replacement by fast MyHCs. However, high frequency neural impulses from the EDL nerve does promote the expression of specific fast MyHC isoforms (Ausoni et al. 1990). The transformation of cross-innervated soleus muscle is incomplete because soleus fibres fail to express 2B MyHC. Note that the neural influences on MyHC expression discussed above take place under a euthyroid state.

The distribution of fibre types in fast and slow muscles of a normal animal is maintained by the neural impulse patterns the fibres receive from the nerve supply (Hennig and Lomo 1985). However, endurance training, by altering the efferent neural impulse patterns can bring about changes towards slower, more oxidative, fatigue resistant fibres. Endurance training in rodents shifts 2b fibres towards 2a/2x fibres in fast muscles (Demirel et al. 1999; Seene et al. 2004; Röckl et al. 2007) and 2a to slow fibres in slow muscle with intense training (Demirel et al. 1999). In horses, endurance training shifts 2x  fibres towards 2a (Rivero et al. 2001) while in humans, this leads to a high proportion of fibres co-expressing 2A and β-slow MyHCs (Klitgaard et al. 1990).

Activity-dependent changes in MyHC gene expression are mediated by variations in the amplitude and duration of the Ca^2+^ transient, which is decoded downstream by Ca^2+^-dependent and other transcription pathways (Gundersen 2011). CLFS induces a sustained but moderately elevated intracellular Ca^2+^ level which activates a Ca^2+^/calmodulin-dependent phosphatase calcineurin which dephosphorylates the nuclear factor of activated T cells (NFATc1), enabling it to enter the nucleus to interact with myocyte enhancer factor 2 (MEF-2C) to induce β-slow MyHC expression (Chin et al. 1998; Naya et al. 2000; McCullagh et al. 2004; Schiaffino et al. 2007; Calabria et al. 2009). Studies using C2C12 myotubes suggest that the transcription factors NFATc1, MyoD, and MEF-2D, as well as the transcriptional coactivator p300 cooperate in Ca^2+^ ionophore-induced upregulation of the β-slow MyHC promoter in a calcineurin-dependent manner (Meissner et al. 2007). At the same time, NFATc1 suppresses fast muscle gene expression by inhibiting the interaction of the nuclear phosphoprotein p300 and MyoD, a crucial regulator of the fast fibre phenotype (Ehlers et al. 2014), facilitating fast-to-slow fibre-type transformation. The increased intranuclear Ca^2+^ level due to CLFS also activates calmodulin-dependent protein kinase that phosphorylates histone deacetylase (HDAC), causing it to exit the nucleus, thereby disinhibiting MEF-2C to induce β-slow MyHC expression (Liu et al. 2005), reinforcing the effect of NFATc1.

The effects of high frequency impulse patterns on MyHC expression may be mediated by their differential effects on the 4 isoforms of NFAT, NFATc1-4 (Calabria et al. 2009). NFAT binding sites are found in the promoter regions of all fast MyHCs and over expression of NFAT preferentially activated 2A MyHC (Allen et al. 2001). Stimulating the EDL with high frequency and high amount stimuli leads to nuclear accumulation of NFATc3, c4 and in some fibres NFATc2. Nuclear localization of NFATc2, c3 and c4 are needed for 2A and 2X MyHC expression, and that of NFATc4 for 2B MyHC expression. CLFS induces the translocation of NFATc1, c2, and c3 into the nucleus of fast fibres already containing NFATc4, thus bringing about the induction of β-slow MyHC expression which requires the nuclear localization of all 4 NFAT isoforms (Calabria et al. 2009).

Thyroid hormone has a profound influence on muscle fibre type and MyHC expression in an apparently muscle specific manner. Early work indicated that thyroid manipulations altered skeletal myosin properties (Ianuzzo et al. 1977). A postnatal surge of thyroid hormone reaching a peak at 2 weeks occurs in the rat, inducing the withdrawal of embryonic and foetal MyHCs in emerging fast and slow fibres and turning on the expression of fast MyHCs (Gambke et al. 1983; Adams et al. 1999), independent of a neural influence (Russell et al. 1988). In the adult rat soleus muscle, hyperthyroidism suppresses β-slow MyHC and induces the expression of 2A and 2X MyHCs but not 2B MyHC (Li and Larsson 1997) while in a fast muscle, hyperthyroidism shifts MyHC expression in the direction 2A→2X→2B (Kirschbaum et al. 1990). Thyroid treatment of cultured myotubes transfected with porcine 2B MyHC gene promotor-reporter construct tended to enhance its expression (Brearley et al. 2021). Hypothyroidism in the soleus muscle causes a 2A→β-slow MyHC change (Nwoye et al. 1982; Diffee et al. 1991b; Caiozzo et al. 1992; Haddad et al. 1997), but in a fast muscle, hypothyroidism causes a 2B→2X→2A shift in MyHC expression that does not proceed to β-slow MyHC expression (Caiozzo et al. 2000; Zhong et al. 2010). However, hypothyroidism does convert a subpopulation of 2b fibres in the superficial white region of a fast muscle into slow fibres without going through 2X and 2A MyHC expression (Zhong et al. 2010).

The mechanism of action of thyroid hormone on MyHC gene expression is highly complex. While it is clear from deletion experiments that thyroid receptors TR-α1 and TR-β are involved (Yu et al. 2000), thyroid response elements (TREs) are not found in the promoter regions for each of the three murine fast MyHC genes (Takeda et al. 1992; Allen et al. 2001), indicating that the hormone acts indirectly to influence fast MyHC expression. In contrast, thyroid hormone stimulates α-cardiac MyHC in the ventricle via a positive TRE and inhibits β-slow MyHC via an inhibitory TRE (Morkin 2000). How thyroid hormone may indirectly regulate fast MyHCs is suggested by the following observations. TREs are found in the promotor regions of the genes for MyoD (Muscat et al. 1994) and myogenin (Downes et al. 1993). These myogenic factors dimerize with ubiquitous E-proteins to bind to specific nucleotide sequences called E-boxes in the regulatory regions of many muscle-specific protein genes, including 2B (Wheeler et al. 1999), 2X and 2A MyHCs (Allen et al. 2001), fast MLC1, MLC3 (Rao et al. 1996) and the intergenic bidirectional promotor between 2X and 2B MyHC genes (Rinaldi et al. 2008), thereby enabling thyroid hormone to regulate their expression via these myogenic factors. This is supported by the following observations: thyroid hormone increases the expression of MyoD in cultured myoblasts and accelerates their differentiation (Carnac et al. 1992). MyoD is expressed in adult fast muscle and myogenin in slow muscle, suggesting that myogenic factors are involved in the specification of adult fibre types (Hughes et al. 1993; Kraus and Pette 1997), and the level of MyoD correlating with their 2B MyHC content. Thyroid induced the appearance of 2X MyHC mRNA and enhancement of 2A MyHC mRNAs in the rat soleus are associated with the appearance of MyoD mRNA (Hughes et al. 1993). The binding of MyoD to an E-box in the 2B MyHC gene is necessary for its expression (Wheeler et al. 1999). Consistent with this, transfection of cultured myotubes with fast MyHC gene promotor-reporter constructs revealed that overexpression of MyoD or Myf-5 preferentially activated the 2B promoter (Allen et al. 2001). Thyroid treatment of cultured myotubes transfected with porcine 2B MyHC gene promotor-reporter construct tended to enhance its expression (Brearley et al. 2021). The expression of specific fast MyHCs is under the control of a super enhancer located between the embryonic and 2A MyHC genes (Dos Santos et al. 2022). The specific transcription factors involved in the thyroid regulation of a single fast MyHC gene in fast fibres have yet to be determined.

The action of thyroid hormone on MyHC expression is modulated by the expression of the thyroid hormone transmembrane transporter, MCT10, which is regulated by the transcription factor sine oculis homeobox1 (Six1) (Girgis et al. 2021). This transcription factor is necessary for the activation of fast muscle program during myogenesis and is more strongly expressed in the nuclei of fast muscle than those in slow muscle, this difference emerging during perinatal growth (Sakakibara et al. 2016). The strong expression of Six1 in the nuclei of fast fibres would thus enhance the intracellular concentration of thyroid hormone via MCT10, increasing MyoD expression and shifting fast MyHC expression in the direction of 2B MyHC while strongly repressing the expression of β-slow MyHC. Knocking out Six1 would be expected to generate an intracellular hypothyroid state in fast fibres, leading to the inhibition of MyoD expression which would inhibit 2B expression and in turn would disinhibit 2X via the 2X anti-sense RNA from the intergenic region between 2X and 2B MyHC genes (Rinaldi et al. 2008). In fact, knocking out Six1 leads to a decrease in 2B and an increase in 2A MyHC expression (Hetzler et al. 2014), simulating the effects of hypothyroidism. In the soleus, Six1 expression is necessary for the expression of 2A MyHC, as Six1 knockout results in the complete loss of 2A and its replacement by β-slow MyHC (Sakakibara et al. 2016). This may be due to the loss of the Six1 regulated linc-RNA and the associated disinhibition of slow-type gene, coupled with the influence of CLFS via the NFAT pathway and the absence of thyroid inhibition of β-slow MyHC due to the low level of MCT10 transporter.

Other hormones also have some influence on MyHC expression. β-adrenergic agonist stimulation has been shown to increase 2B MyHC and decrease 2A MyHC expression in somitic muscles of sheep (Hemmings et al. 2015) and pig (Gunawan et al. 2007, 2020), while growth hormone has no influence on MyHC expression (Hemmings et al. 2015). β-adrenergic agonist stimulation also induces de novo expression of 2X MyHC in rat soleus (Oishi et al. 2002). Although some sexual differences in fibre-type distribution have been described in limb muscles of experimental animals and humans, there is little evidence that sex hormones directly influence fibre-type change in these muscles (Haizlip et al. 2015). However, testosterone influences MyHC gene expression in rodent jaw-closing but not limb muscles, shifting 2a fibres towards 2b fibres in the mouse (Eason et al. 2000) and guinea pig (Lyons et al 1986) and shifting α-cardiac fibres towards 2a fibres in the rabbit (Reader et al. 2001). This illustrates the allotypic difference between jaw and limb muscles in rodents.

To acquire a coherent view of the regulation of MyHC expression in muscle fibres, it is important to appreciate that thyroid hormone and neural impulse patterns mutually oppose each other in their influences on MyHC expression. Thyroid hormone shifts MyHC expression in fast muscle in the direction 2A→2X→2B, while CLFS opposes this (Kirschbaum et al. 1990), rapidly eliciting a 2B→2X→2A transition of MyHCs in the euthyroid state, leading to the total absence of 2B MyHC while 2A and 2X MyHCs predominated, but fails to induce β-slow MyHC within 2 months (Ausoni et al. 1990). In the hypothyroid state, however, CLFS does induce the β-slow MyHC (Kirschbaum et al. 1990) and this induction of β-slow MyHC expression is suppressed in the hyperthyroid animal (Zhou et al. 2019).

Thyroid hormone may inhibit β-slow MyHC expression indirectly by activating the expression of miR-133a1, which is enriched in fast muscle (Zhang et al. 2014). miR-133a1 inhibits a TEA family member 1 (TEAD1) by binding to the 3’ UTR of its mRNA (Zhang et al. 2014). TEAD1 regulates the transcription of β-slow MyHC (Tsika et al. 2008), so thyroid induction of miR-133a1 leads to the inhibition of β-slow MyHC expression. Knockdown of TEAD1 decreases the expression of β-slow MyHC and increased the expression of fast MHCs, while overexpression of miR-133a1 in the soleus mediates a slow-to-fast fibre-type transition (Zhang et al. 2014). In the ventricular myocardium, thyroid hormone induces the inhibition of β-slow MyHC (Hoh et al. 1978) via thyroid receptor TRα1 (Mansen et al. 2001) acting on a negative TRE in the regulatory region of the β-slow MyHC gene (Morkin 2000). However, transfection studies in C2C12 myoblasts suggest that this pathway is inoperative in skeletal muscle (Zhang et al. 2014).

The molecular mechanism whereby CLFS opposes the thyroid influence on fast MyHC expression is that the increased nuclear NFATc1 in response to CLFS of fast fibres inhibits MyoD-dependent fast fibre gene promoters by physical interaction of NFATc1 with MyoD, thereby blocking the recruitment of the essential transcriptional coactivator p300 needed for the expression of fast MyHCs and other fast muscle genes (Ehlers et al. 2014). This is consistent with the observation that transfection of a constitutively active mutant NFATc1 in the rat EDL inhibits the 2B MyHC promoter (McCullagh et al. 2004). Earlier work had shown that CLFS of a fast muscle leads to deactivation of MyoD by phosphorylating it and inhibiting its expression (Ekmark et al. 2007). This inhibition of MyoD reinforces the action of NFATc1 in inhibiting the action of thyroid hormone on fast MyHC expression.

The above observations indicate that changes in MyHC gene expression in response to neural and thyroid hormone stimulation are muscle-specific and have been reported by many authors (Izumo et al. 1986; Gustafson et al. 1986; Fitzsimons et al. 1990; Jakubiec-Puka et al. 1999). These differences have hitherto been attributed to different adaptive ranges of fast and slow muscles to various stimuli (Westgaard and Lomo 1988). However, detailed analyses below of neural and thyroidal influences on muscle fibres of different developmental origins in the light of the mutual antagonistic interactions between these influences reveal that these differences in adaptive range arise from (1) the intrinsic characteristic properties of fibres derived from different lineages of primary and secondary myotubes, and (2) the interactions between the thyroid state and the diverse neural impulse patterns muscle fibres receive. Fibres of different developmental origins may end up with the same phenotype at maturity. Fibres of a given phenotype may respond differently to neural or thyroidal stimulation depending on their developmental origins. It is thus important to classify muscle fibres in mature animals not only according to their phenotype, but also according to their developmental origin, which will be referred to below as their ontotype.

Three hierarchical terms in relation to fibre types are used in this review: allotype, ontotype and phenotype. Allotype is an attribute of a muscle developing from a distinct region of the body with distinct functional demands. The cellular basis for muscle allotype is the diversity of myogenic progenitor cells in these regions which are committed to myogenesis by different regulatory factors. The repertoire for MyHC expression in the fully differentiated fibres of each muscle allotype is generally very different from that of other allotype and are subject to adaptive changes in response to changes in functional demands during phylogeny. The term ontotype refers to the diverse types of primary and secondary myotubes that emerge during myogenesis of a muscle which respond differently to postnatal neural and thyroidal influences to generate fully differentiated fibre phenotypes. Fibres of a given phenotype may arise from myotubes of different ontotypes, but they retain their capacity to respond differently to neural and thyroidal influences during postnatal life.

### Diversity of somitic myotubes, fibre types they give rise to, and their responses to neural and thyroidal influences

During developmental myogenesis of somitic muscles, MyHC expression is influenced by the lineage of myoblast forming the myotube as well as by hormonal and neurogenic mechanisms. The role of myoblast lineage in influencing MyHC gene expression during myogenesis in the chick was established by the pioneering work of Stockdale and co-workers (Miller and Stockdale 1986; Stockdale 1992). In mammals, two distinct myoblast lineages with different patterns of gene expression during myogenesis emerge sequentially from the somites: embryonic and foetal myoblasts (Cossu and Biressi 2005; Hutcheson et al. 2009). Purified embryonic and foetal myoblasts during myogenesis in culture constitutively express genes characteristic of slow and fast fibres respectively (Biressi et al. 2007). Embryonic myoblasts migrate into the limb bud and fuse with one another to form primary myotubes which are later surrounded by secondary myotubes formed by the fusion of mainly foetal myoblasts. The formation of secondary myotubes is dependent upon functional innervation of primary myotubes which is necessary for foetal myoblast maturation (Harris 1981; Ross et al. 1987; Wilson and Harris 1993).

Primary myotubes express embryonic and β-slow MyHCs (Narusawa et al. 1987) while secondary myotubes express embryonic and neonatal MyHCs but not β-slow MyHC (Pin and Merrifield 1993; Ghosh and Dhoot 1998). Embryonic myoblasts express Sox6, a member of the Sox transcription factor family, which turns on the expression of MEF-2C, inducing the expression of β-slow MyHC (Hagiwara et al. 2007; Taglietti et al. 2016). Foetal myoblasts strongly express the transcription factor nuclear factor one X (Nfix) which acts as a main regulator that converts embryonic myoblasts into foetal myoblasts (Messina et al. 2010). Nfix reverses the function of Sox6 from stimulation of β-slow MyHC expression in primary myotubes to one of inhibition in secondary myotubes by forming a complex with Sox6 and binds to an inhibitory site in the promoter region of β-slow MyHC gene, suppressing β-slow MyHC expression (Taglietti et al. 2016). Analyses of the promotor regions of embryonic and foetal MyHC genes suggest that myogenic factors and the calcineurin pathway are involved in their regulation (Beylkin et al. 2006). Analysis of mouse mutants shows that MyoD and NFAT2c collaborate to express neonatal MyHC during perinatal development (Daou et al. 2013).

The activation of fast muscle program during myogenesis depends on the transcription factors Six1 and Six4 (Niro et al. 2010; Richard et al. 2011; Wu et al. 2014). Earlier work has shown that the forced expression of Six1 and cofactor Eya1 in the adult slow-twitch soleus muscle induces a fibre-type transition characterized by the replacement of β-slow and 2A MyHCs with 2B and/or 2X isoforms, the activation of other fast-twitch fibre-specific genes and a switch toward glycolytic metabolism (Grifone et al. 2004). Six1 controls the expression of a long intergenic non-coding RNA (linc-RNA) gene located between embryonic and 2A MyHC genes in the fast MyHC gene cluster. Expression of linc-RNA is confined to fast muscle fibres, where it suppresses β-slow MyHC expression. The Six1 regulated enhancer of this linc-RNA gene is shared by the three fast MyHC genes, enabling Six1 to promote the expression of fast MyHC genes while inhibiting β-slow MyHC (Sakakibara et al. 2014), thus stabilizing the fast fibre phenotype.

During postnatal myogenesis, MyHC expression of all myotubes come under the simultaneous influences of a postnatal surge of thyroid hormone (Gambke et al. 1983; d'Albis et al. 1987b) and the emerging CLFS or high frequency neural impulse patterns from their nerve supplies. In the rat, the thyroid surge reaches a peak at 2 weeks, inducing the withdrawal of embryonic and foetal MyHCs in emerging fast and slow fibres and turning on the expression of fast MyHCs in developing fast muscles (Gambke et al. 1983; Adams et al. 1999) apparently independent of a neural influence (Russell et al. 1988). Hypothyroidism in the neonate delays fast MyHC expression (Butler-Browne et al. 1984; Mahdavi et al. 1987) while hyperthyroidism accelerates it (d'Albis et al. 1987a).

Muscle fibres in the neonate are initially polyneuronally innervated, this situation is eliminated in rodents by the first two weeks of life (Redfern 1970; Personius and Balice-Gordon 2001), enabling different patterns of fast and slow motoneuron activities (Hennig and Lomo 1985) to be delivered to specific populations of myotubes to generate the emerging fast and slow motor units. The integrity of the nerve is crucial to the expression of β-slow MyHC in emerging slow fibres (Gambke et al. 1983; d'Albis et al. 1987b; Adams et al. 1999).

At least four types of myotubes can be defined according to the developmental origin of their myoblasts and the mature fibre type they give rise to: slow primaries, fast primaries, slow secondaries, and fast secondaries. These four types of myotubes have unique properties and respond differently to the developmental surge of thyroid hormone depending on the pattern of nerve impulses they receive. Both primary and secondary myotubes may develop into phenotypically slow or fast fibres at maturity (Hoh et al. 1988b; Harris et al. 1989).

In the neonatal rat soleus muscle, three types of myotubes emerge, slow primaries, slow secondaries, and fast secondaries. The slow primary myotubes express embryonic and β-slow MyHCs early postnatally, the expression of the latter is sustained by the Sox6/MEF-2C pathway (Taglietti et al. 2016). Slow primaries continue to express β-slow MyHC later during development and at maturity via the calcineurin/NFAT pathway (Oh et al. 2005), being driven by CLFS from the soleus nerve. Following eight weeks of deprivation of CLFS by denervation of adult rat soleus, a large population of fibres retain the expression of β-slow MyHC (Hugh and Hoh 1987; Gorza et al. 1988), consistent with the preservation of the bulk of β-slow MyHC in myosin extract from denervated rat soleus (Hämäläinen and Pette 1996). Persistence of slow fibres following denervation has also been reported in the mouse (Agbulut et al. 2009) and rabbit (d'Albis et al. 1994) soleus. Since it is known that β-slow MyHC expression during embryonic myogenesis is independent of CLFS and acts via the Sox6/MEF-2C pathway (Taglietti et al. 2016), it is likely that fibres persisting to express β-slow MyHC following denervation are of slow primary ontotype (and slow secondary ontotype which also express β-slow MyHC as myotubes) and are reverting to their embryonic characteristic. The expression of some embryonic MyHC in denervated slow muscle (Hamalainen and Pette 1996; Haddad et al. 1997) supports this view. It may be argued that the persistence of β-slow MyHC expression following denervation of the soleus is induced by fibrillation potentials which have a mean frequency of 4.5 Hz (Purves and Sakmann 1974) acting similarly to CLFS. This is unlikely since, in the denervated EDL, only fibres of slow primary origin express β-slow MyHC (Hoh 1991) even though fibrillation potentials are not fibre-type specific (Purves and Sakmann 1974).

Hyperthyroidism, which induces MyHC expression in a fast muscle in the direction 2A→2X→2B (Kirschbaum et al. 1990), induces the expression of 2A and 2X MyHCs in the adult soleus, but not 2B MyHC (Hämäläinen and Pette 1996; Li and Larsson 1997). This failure to induce 2B MyHC applies to slow fibres of all soleus ontotypes and is likely due to an inhibitory effect of CLFS from the soleus nerve on the thyroid influence on 2B MyHC expression. CLFS shifts fast MyHC expression in the direction 2B→2X→2A in a fast muscle (Termin et al. 1989; Ausoni et al. 1990). This appears to be brought about by NFATc1 induced by CLFS inhibiting the 2B MyHC promoter (McCullagh et al. 2004), Hyperthyroidism does robustly induce 2B MyHC expression in the soleus of hindlimb suspended rats (Haddad et al. 1998) in which CLFS is lost. 2B MyHC expression in this situation is inhibited by denervation (Di Maso et al. 2000), indicating that some neural activity is necessary. This may be the high frequency stimuli, which induces 2X and 2B MyHC expression in denervated soleus muscle (Hämäläinen and Pette 1996). This is supported by the observation that the chronic low frequency impulse pattern of slow motoneurons in hindlimb suspended rats is transformed to one resembling that of fast motoneurons (Cormery et al. 2005). Future work is needed to confirm this possibility and to determine the soleus ontotypes in which 2B MyHC expression occurs under hyperthyroidism plus hindlimb suspension, and to establish the mechanism thereof.

The slow secondary myotubes of the soleus form late in gestation and express embryonic, neonatal, and β-slow MyHCs, ending up continuing the expression of β-slow MyHC in the mature muscle from receiving CLFS from the soleus nerve. Depriving soleus fibres of CLFS by cordotomy, cross-innervation by a fast muscle nerve (Hoh et al. 1980) and spinal cord isolation (Talmadge 2000; Huey et al. 2001) removes the inhibitory effect of CLFS on thyroid influence on fast MyHC expression, enabling soleus fibres to respond to the euthyroid state by expressing 2A and 2X MyHCs. Fibres responding this way are most likely of slow secondary ontotype, which are the most abundant in the rat soleus. Removing CLFS by denervation of the soleus also leads to a substantial increase in the content of 2A and 2X MyHCs at the whole muscle level (Hämäläinen and Pette 1996). Hyperthyroidism, by opposing CLFS, reduces the content of β-slow MyHC and increases the content of 2A and 2X MyHCs to 34% of MyHC pool (Haddad et al. 1997), while denervation plus hyperthyroidism further increases these isoforms (Haddad et al. 1997). All single soleus fibres from hyperthyroid rats co-express β-slow and 2A or 2A/2X MyHCs (Li and Larsson 1997), indicating that slow fibres of both slow primary and slow secondary ontotypes are involved. The rational for the failure of thyroid hormone to induce 2B MyHC is that CLFS opposes the thyroid influence, as discussed above in connection with soleus slow primaries.

The fast secondaries of the soleus, which form during early postnatal life, express embryonic and neonatal MyHCs but not β-slow MyHC and end up expressing 2A MyHC at maturity (Butler-Browne and Whalen 1984; Narusawa et al. 1987). 2A MyHC expression depends on the neonatal surge of thyroid hormone, since following hypothyroidism, soleus 2a fibre with intact innervation are replaced by slow fibres (Nwoye et al. 1982; Caiozzo et al. 1992; Vadaszova et al. 2004). This is likely due to the CLFS from the soleus nerve. This response differs from that of fast secondaries of the EDL in which hypothyroidism does not induce β-slow MyHC expression in the absence of CLFS.

Soleus fibres of fast secondary origin do not express 2X and 2B MyHCs under thyroid influence probably because of opposing action of low frequency impulses from the slow motor nerve which gradually transform them into slow fibres as the rat grows older (Kugelberg 1976; Butler-Browne and Whalen 1984), presumably due to impulse patterns becoming increasingly close to CLFS in response to increasing functional load as body mass increases. The slow fibres of the soleus that become pure 2a fibres following long term denervation (Hugh and Hoh 1987) most likely represent a reversal of the age-dependent 2a-to-slow change (Kugelberg 1976; Butler-Browne and Whalen 1984) in fibres of fast secondary ontotype. With the loss of CLFS, these fibres now respond to the euthyroid state by expressing 2A MyHC. During hyperthyroidism, these fibres do not express 2B MyHCs, but they probably do with hyperthyroidism plus hindlimb suspension, as discussed above in connection with slow primary ontotype.

In developing rat fast muscles such as the tibialis anterior and EDL, there are three types of myotubes: slow primaries, fast primaries, and fast secondaries. Slow primaries are found in the deep, red region of the muscle, each slow primary fibre being surrounded by a rosette of fast secondary myotubes (Narusawa et al. 1987). The slow primaries express embryonic and β-slow MyHCs early postnatally, as in slow primaries of the soleus. The β-slow MyHC expression is presumably sustained by Sox6/Mef-2C pathway (Taglietti et al. 2016). All slow primaries of the rat EDL upon neonatal denervation continue to express β-slow MyHC and resists atrophy, in contrast to their atrophic neighbouring fast fibres of fast secondary ontotype (Hoh et al. 1989; Russell et al. 1993).

A large proportion of slow primaries in developing fast muscles undergo β-slow→2A MyHC transformation during postnatal development (Narusawa et al. 1987). This results from a lack CLFS from the fast muscle nerve to continue β-slow MyHC expression. These fibres now respond to the postnatal surge in thyroid hormone to express fast MyHCs but presumably also receive the high frequency, high amount stimuli from the EDL nerve favouring the expression of 2A MyHC (Ausoni et al. 1990). Following denervation of the EDL in the adult, the number of slow fibres in the red region of the muscle progressively increase to about the number of slow primaries in the newborn animal by the reversal of the β-slow→2A MyHC transformation, the full complement of these slow fibres resists denervation atrophy (Hoh 1991). At the whole muscle level, these changes are reflected by the doubling of the β-slow MyHC and increased 2A MyHC content (Haddad et al. 1997). Hypothyroidism leads to an increase in β-slow MyHC content (Jakubiec-Puka et al. 1999) and fast-to-slow fibre-type transformation (Vashishta and Talesara 2000) in fast muscle, reflecting the reversal of the β-slow→2A MyHC transformation. It is hypothesized that expression of β-slow MyHC in the absence of CLFS in denervated fast muscle and in hypothyroidism is due to the reversion to the mechanism of β-slow MyHC expression of slow primary myotubes sustained by Sox6/MEF-2C pathway (Taglietti et al. 2016), in common with soleus fibres of slow primary ontotype.

Fast primary myotubes in rat fast muscles are confined to the superficial, white region and are surrounded by fast secondary myotubes (Dhoot 1986; Narusawa et al. 1987; Condon et al. 1990). Fast primaries perinatally express embryonic, neonatal, and β-slow MyHCs (Harris et al. 1989) and these MyHCs are progressively replaced by 2B MyHC during postnatal life (Zhang and McLennan 1998), there being no slow fibres in the white region of adult fast muscles. In sharp contrast to slow primaries in both fast and slow muscles, fast primary myotubes of euthyroid rat EDL do not express β-slow MyHC after neonatal denervation (Hoh et al. 1989), but most likely express 2B MyHC. This is supported by the observation that in the neonatally denervated rat EDL, a population of myotubes located in the superficial region replaces neonatal MyHC with 2B MyHC (Russell et al. 1993). This developmental expression of 2B MyHC is likely a response to the neonatal surge of thyroid hormone, since hypothyroidism in the adult uniquely suppresses 2B MyHC expression in fibres of fast primary ontotype, replacing it with β-slow MyHC (Zhong et al. 2010), as the inhibitory action of thyroid hormone on β-slow MyHC gene is removed. The expression of β-slow MyHC in the absence of CLFS is likely to be due to a reversion to β-slow MyHC expression of fast primary myotubes (Harris et al. 1989) and is likely to be sustained by Sox6/MEF-2C pathway (Taglietti et al. 2016). In contrast, neighbouring 2b fibres of the fast secondary ontotype undergo a 2B→2X→2A MyHC shift during hypothyroidism (Zhong et al. 2010).

Fast secondary myotubes in developing fast muscles express embryonic and neonatal MyHCs but not β-slow MyHC (Harris et al. 1989) in common with fast secondaries of the soleus, but they end up as 2a, 2x  and 2b fibres in response to the thyroid surge and being modulated by high or low amounts of the high frequency impulse patterns from the EDL nerve (Ausoni et al. 1990) into the various fast fibre types. The thyroid surge would induce fast MyHC expression in the direction 2A→2X→2B, tending to suppress 2A MyHC expression (Kirschbaum et al. 1990), but this is opposed by high frequency, high amount stimuli (Ausoni et al. 1990), which favours 2A MyHC expression. Neonatal denervation, by removing high frequency, high amount stimuli, limits the response to the thyroid surge in these fibres to 2B and 2X MyHCs, to the exclusion of 2A MyHC (Russell et al. 1993). This inhibition of 2A MyHC expression following neonatal denervation may be due to an antisense transcript of 2A MyHC gene associated with enhanced transcription of the 2X MyHC gene, as observed in slow muscle following spinal cord isolation (Pandorf et al. 2006). Denervation of the adult EDL, however, showed no loss of 2A MyHC expression (Huey and Bodine 1998), the mechanism for 2A MyHC expression here is not known. Fibres of fast secondary origin are the most abundant in a fast muscle and can be transformed into slow fibres by CLFS of long duration (Windisch et al. 1998) or cross-innervation by a slow nerve (Hoh et al. 1980), just as 2a fibres of fast secondary ontotype in the soleus transforming into slow fibres with advancing age (Kugelberg 1976).

Hypothyroidism in an adult fast muscle shifts fast MyHC expression in fibres of fast secondary ontotype in the direction 2B→2X→2A (Kirschbaum et al. 1990), resulting in the accumulation of 2a fibres which do not progress to expressing β-slow MyHC (Zhong et al. 2010). In contrast, soleus fibres of fast secondary ontotype do express β-slow MyHC in hypothyroidism (Nwoye et al. 1982; Diffee et al. 1991b; Caiozzo et al. 1992; Haddad et al. 1997). This can be explained by their difference in innervation: the soleus fast secondaries receive tonic low frequency impulses from its slow nerve which promotes β-slow MyHC expression, whereas the EDL fast secondaries receive intermittent high-frequency impulses, which promotes fast MyHC expression (Ausoni et al. 1990).

The above discussion shows that fibres derived from the four types of myotubes have distinct intrinsic properties. The differences in adaptive ranges of whole fast and slow muscles are attributable to the adaptive ranges of their component ontotypes. For each ontotype, the phenotypic outcome at maturity under the same thyroid influence differs depending on the impulse pattern delivered by the innervating motoneuron. The apparent complexity of the thyroidal influence on MyHC expression in fast and slow muscles (Fitzsimons et al. 1990; Jakubiec-Puka et al. 1999) becomes coherent when fibre ontotypes and neural impulse patterns are taken into consideration. Recognizing that different ontotypes within fast or slow muscles have distinct properties, future work on factors that influence MyHC gene expression should include immunohistochemistry rather than whole muscle biochemistry alone.

During myogenesis, a third type of cell with myogenic potential emerges and is maintained in an undifferentiated state as satellite cells in the adult animal, lying between the basal lamina and the fibre plasma membrane of fast and slow fibres (Cossu and Biressi 2005; Rodriguez-Outeiriño et al. 2021). During postnatal development and growth, satellite cells contribute new nuclei to the growing muscle fibre. In the mature animal, satellite cells divide at a slow rate and a large part of the progeny fuse with the subjacent fibre to contribute new nuclei to the growing muscle fibre. Satellite cells serve as muscle stem cells which are activated to execute the myogenic program during muscle growth and regeneration in response to tissue damage.

Satellite cells of muscles of different allotype are distinct lineages committed to express the MyHC repertoire of the allotype (Hoh and Hughes 1991; Harel et al. 2009; Hoh 2021). Satellite cells in somitic muscle are heterogeneous in their commitment to MyHC expression. Those isolated from single slow fibres form a considerably higher proportion of myotubes expressing β-slow MyHCs in tissue culture than satellite cells from fast fibres (Rosenblatt et al. 1996; Bryla and Karasinski 2001). The notion that there are distinct lineages of satellite cells in somitic muscles is supported by regeneration studies on rat soleus and EDL muscles. Stimulation of regenerated fibres of rat soleus and EDL muscles with the same tonic impulse pattern shows that all regenerated soleus fibres express β-slow MyHC, while the regenerated EDL has only scattered single or small groups of fibres expressing β-slow MyHC (Kalhovde et al. 2005).

#### The origin of ontotype heterogeneity

The different responses of fibres of diverse developmental origins to neural and thyroidal stimulation must have cellular and molecular bases. Two hypotheses have been proposed to explain the diversity of adaptive range among muscle fibre types. Parry suggested that embryonic myoblasts can only express β-slow and 2A MyHCs during myogenesis while foetal myoblasts can only express 2A, 2X and 2B MyHCs. Developing myotubes are formed by the fusion of different proportions of primary and secondary myoblasts (Parry 2001). Thus, myotubes of the soleus originating from predominantly embryonic myoblasts would be able to express only β-slow and 2A MyHCs.

Pin et al. (2002) showed that myoblasts can be divided into three types according to the pattern of MyHC expression during in vivo myogenesis after transplanting myoblasts into brain tissue: embryonic myoblasts that express β-slow MyHC, fast foetal myoblasts that express fast MyHCs and fast/slow foetal myoblasts that express β-slow/fast MyHCs. Embryonic myoblasts give rise to primary myotubes which mature predominantly into slow fibres, while fast foetal myoblasts form secondary myotubes that mature into fibres having the adaptive range 2A ↔ 2X ↔ 2B, and fast/slow foetal myoblasts give rise to fibres with adaptive range of β-slow ↔ 2A.

Neither hypothesis encompasses the existence of the fast primary ontotype, nor provide any molecular mechanism, but both hypotheses do emphasize the importance of the composition of myonuclei in the developing myotubes. Both embryonic and foetal myoblasts can fuse into primary and secondary myotubes (Evans et al. 1994; Zhang and McLennan 1995; Wigmore and Evans 2002) and satellite cells are incorporated into myotubes from early postnatal life. The interactions of myonuclei from different sources may generate stable and unique patterns of transcription factors that modulate the properties of different ontotypes. A possible example of the molecular basis for the resistance of fast secondary ontotype to express β-slow MyHC during hypothyroidism (Caiozzo et al. 2000; Zhong et al. 2010) and following CLFS (Ausoni et al. 1990) is as follows. As described above, thyroid hormone inhibits β-slow MyHC via the induction of miR-133, which inhibits TEAD1, a regulator of β-slow MyHC. This inhibitory pathway may be fortified in fast fibres by the high expression of Six1 in nuclei of fast muscle compared with slow muscle (Sakakibara et al. 2016), inducing the expression of MCT10, the thyroid hormone transporter (Girgis et al. 2021). Fast fibres may thus retain a moderate intracellular level of thyroid hormone during hypothyroidism to sustain the inhibition of β-slow MyHC, explaining the difficulty of inducing β-slow MyHC expression during hypothyroidism and following CLFS in fibres of the fast secondary ontotype. Future research should attempt to find markers for the various fibre ontotypes and address the prevailing network of transcription factors in fibres of various ontotypes and the mechanisms whereby they respond differently to the same physiological stimuli.

#### Influence of body size on MyHC isoforms expressed in somitic muscles

The myosin isoforms expressed in mammalian limb and trunk muscles are influenced by the animal’s body size. The full complement of four fibre types is present in eutherian mammals of small body size (e. g. mouse, rat, and guinea pig) (Termin and Pette 1991; Hamalainen and Pette 1993; Rivero et al. 1998). In very small mammals, e. g. shrews, slow fibres in postural muscles are completely replaced by fast ones (Savolainen and Vornanen 1995; Peters et al. 1999). MyHC 2B isoform is not expressed in limb muscles of most eutherians of intermediate and large size, such as cats (Talmadge et al. 1996; Lucas et al. 2000), dogs (Smerdu et al. 2005), primates (Smerdu et al. 1994; Ennion et al. 1995; Lucas et al. 2000), cattle (Maccatrozzo et al. 2004; Toniolo et al. 2005) and horses (Rivero et al. 1999; Chikuni et al. 2004). In sheep and cattle, 2B MyHC is normally weakly expressed at the mRNA level but rarely or not at all at the protein level (Hemmings et al. 2009; Picard and Cassar-Malek 2009). The absence of 2b fibres in these eutherians means that these animals use 2x fibres for fast locomotion and will benefit from their oxidative metabolism and higher endurance to enable them to run long distances without fatigue, possibly also in enhancing the saving of elastic energy during locomotion (see later). However, expression of 2B MyHC isoform is found in locomotory muscles of the llama (Graziotti et al. 2001) and the pig (Lefaucheur et al. 1998; Toniolo et al. 2004). The expression 2B MyHC can be induced in sheep (Hemmings et al. 2015) by a β-adrenergic agonist, an agent which enhances 2B MyHC expression in the pig (Gunawan et al. 2007). There is a 3 bp difference within the CArG-box region of the 2B MyHC gene promoter between pig and human that may explain the failure of 2B MyHC expression in humans, but there is no common mutation among other animals that do not express 2B MyHC (Brown et al. 2014). The variations in MyHC expression in limb muscles across species reflect the plasticity of muscle at the phylogenetic level. The molecular basis for the loss of 2B MyHC expression in some mammals needs clarification by future work.

Unlike eutherian mammals, large and small marsupial mammals express the full complement of limb MyHCs (Sciote and Rowlerson 1998; Zhong et al. 2001, 2008, 2010). Remarkably, there is little or no 2B MyHC expression in the gastrocnemius and flexor digitorum profundus muscles in hopping marsupials, despite the predominance of 2b fibres in most of their other hindlimb muscles (Zhong et al. 2008). These muscles are involved in elastic energy saving during hopping (Biewener et al. 1998). In sharp contrast, non-hopping marsupials strongly express 2B MyHC in their gastrocnemius muscle (Zhong et al. 2008). It is not known whether this highly specific suppression of 2B MyHC expression in hopping marsupials originates during developmental myogenesis or induced by hopping after pouch exit. The mechanism for this suppression of 2B MyHC expression in marsupial muscles involved in elastic energy saving is interesting and well worth prompt investigation.

#### MyHC expression and adaptations to hopping and elastic energy saving

The hopping of macropodids requires powerful hip and knee extensors to generate an acceleration of about 3–4 g during take-off compared with up to 1.7 g for animals that run (Cavagna et al. 1988). These extensor muscles have very high contents (35–87%) of 2b fibres (Zhong et al. 2008) to provide the necessary power. This may be an important factor involved in the preservation of 2b fibres in macropods in contrast to loss of 2b fibres in many large eutherians.

The functional significance of the virtual absence of 2b fibres specifically in the ankle and digital extensors of hopping marsupials and the near global loss of 2b fibres of limb and trunk muscles in most large eutherians studied may be related to the saving of elastic energy during locomotion (Alexander 1984, 2002). As a leg strikes the ground during a hop or run, kinetic and gravitational potential energy is temporarily stored as elastic strain energy in the stretched muscles and their tendons and is then recovered during the propulsive phase of the hop or step. Muscles involved need to contract isometrically. Stretching an isometrically contracting muscle leads to an increase of force (Katz 1939) which has been attributed to the recruitment of cross-bridges that exhibit fast attachment to, and detachment from, actin in a cycle that does not involve ATP splitting (Linari et al. 2004). This leads to the absorption of work, which is reflected by reduced ATP utilization (Infante et al. 1964). The 2x fibres may be able to conserve more ATP in this situation than 2b fibres. Further, use of the slower 2x fibres involves less energy expenditure than 2b fibres to produce the same isometric force. The stiffness of 2X MyHC cross-bridges is three times higher than that of β-slow MyHC (Percario et al. 2018) and may also be higher than that of 2B MyHC. A higher muscle stiffness would allow more elastic energy to be stored in the muscle as well as in the tendon, though most of the energy is stored in the tendons. Elastic energy storage and recovery as a means of conserving energy during locomotion occurs in both large eutherian and marsupial mammals (Biewener 1998; Alexander 2002). The fact that in eutherians this involves muscles of both forelimbs and hindlimbs (Biewener et al. 1998) may require extensive use of 2x fibres in all limbs, thus helping to explain their global loss of 2b fibres.

#### Influence of body size on the kinetics of myosin isoforms

Analysis of mechanical properties of whole muscles showed that the Vmax of fast and slow muscles of different species is inversely related to body size (Close 1972) and can be expressed as a power function of body mass with a negative exponent. Rome et al. showed that Vmax of slow fibres of rat, rabbit, and horse scale with a body mass exponent of -0.18 but the value for fast fibres was only -0.07 (Rome et al. 1990). The greater influence of body mass on the Vmax of slow fibres was confirmed by Pellegrino et al. who reported a body mass exponent of -0.175 for slow fibres, but -0.041 for 2b fibres, -0.048 for 2 × fibres, -0.098 for 2a fibres (Pellegrino et al. 2003). Marx et al. showed that in animal species that cover a 100,000-fold range of body mass, Vmax varies with a mass exponent of -0.08 for slow fibres and -0.04 for 2a fibres (Marx et al. 2006) but varies more strongly as a function of femur-length, with an allometric exponent of -0.23 for slow fibres and -0.14 for 2a fibres (Marx et al. 2006). Medler showed that for terrestrial animals Vmax of muscle fibres scales with a body mass exponent of -0.25, but for 72 species of animals ranging from the fruit fly to cattle with a mass exponent of -0.17 (Medler 2002).

The scaling of Vmax of MyHCs on body mass has an impact on the energy cost of locomotion in animals of different body size. The metabolic energy consumed by each gram of an animal, as it moves along the ground at a constant speed, increases linearly with speed and is proportional to (body mass)^−0.3^ (Heglund et al. 1982). It has been suggested that the metabolic cost of constant speed locomotion is due to the cost of generating force by the muscles (Taylor et al. 1980), which in turn is related to the Vmax of active muscle fibres. The modest negative body mass exponent of Vmax for MyHCs cited above (-0.175 to -0.098) goes only part of the way to account for the body mass exponent of -0.3 for metabolic cost of locomotion. The rest of the energy cost may lie in the cost of muscle activation, which is higher in small animals than in large. Small animals have higher stride frequencies (Heglund et al. 1974) and turning their muscles on and off by the release and reuptake of Ca^2+^ would incur much more energy than in large animals.

The allometric exponents for the Vmax of β-slow MyHC relative to other MyHCs cited above indicate that body mass has a greater influence on the kinetics of β-slow MyHC. This may be related to its function in the heart. Heart rate scales to (body mass)^−0.25^ (Stahl 1967) and the kinetics of cardiac contraction must be similarly scaled for proper function. This is partly achieved by the scaling of the Vmax of β-slow MyHC, the dominant ventricular myosin in large animals, to around (body mass)^−0.18^ and by shifting the expression of ventricular MyHC to the faster α-cardiac MyHC in small eutherians (Lompre et al. 1981; Kessler-Icekson et al. 1988) as well as small marsupials (Hoh et al. 2007a). The changes in kinetics of β-slow MyHC of animals over a wide range of body masses are accomplished by mutations confined principally to 12 sites in the amino acid sequence of β-slow MyHC (Johnson et al. 2021).

### Overview and functional significance of mechanisms regulating MyHC expression

This review has highlighted the fact that the pattern of expression of MyHCs in mammals is principally influenced by the complex interplay of cell lineages, neural impulse pattern, thyroid hormone, body mass and functional demand. Even before myogenesis, myoblast progenitors of craniofacial and somitic muscles from different regions of the mesoderm are channelled into specific lineages of progenitor myoblasts and satellite cells that give rise to the various muscle allotypes with different repertoires for MyHC expression during subsequent myogenesis. During somitic muscle myogenesis, myoblast progenitors that give rise to embryonic and foetal myoblast lineages emerge and form slow and fast primary and secondary myotube ontotypes which give rise to slow and various fast muscle fibre types. These ontotypes determine the topographic distribution of the fibre types within the muscle and their specific modes of response to neural and hormonal influences.

During early postnatal life, the emerging myotubes simultaneously come under the influences of a surge of thyroid hormone and the different impulse patterns of fast and slow motoneurons. The surge of thyroid hormone stimulates the expression of fast MyHCs in fast muscles to enhance speed and power. Simultaneously, high frequency neural impulses from fast motoneurons modulate the expression of different fast MyHC isoforms to give rise to the various fast fibre types. In slow muscles, the slow motoneurons and the neonate’s encounter with gravity generate tonic low frequency neural impulses which induce β-slow MyHC in postural muscle fibres.

The postnatal surge of thyroid hormone enhances cellular metabolism (Hulbert 2000) to support the rapid growth and increased physical activity of the neonate. It also induces changes in mammalian cardiac MyHC expression, inducing α-cardiac MyHC expression in the ventricle while suppressing β-slow MyHC (Hoh et al. 1978; Lompre et al. 1981; Kim et al. 2007), enhancing cardiac contractility (Rossmanith et al. 1986) to meet the thyroid induced enhanced metabolism. Thyroid hormone also enhances 2B MyHC expression in laryngeal muscles (Wu et al. 2000a), the fast kinetics of this isoform helping to cope with the increased respiratory frequency associated with the thyroid induced enhanced metabolism.

Body mass strongly influences physiological rate processes. McMahon, arguing theoretically from elastic similarity, concluded that metabolic rate should scale to (body mass)^0.75^ and physiological cycles should scale to (body mass)^−0.25^ (McMahon 1973). Experimentally determined basal metabolic rates in eutherian and marsupial mammals do scale approximately to (body mass)^0.75^ (Kleiber 1961; Dawson and Hulbert 1970; Schmidt-Nielson 1984; Savage 2004), the specific metabolic rate per unit body mass thus scales to (body mass)^−0.25^. Heart rate and respiratory frequency are similarly scaled to (body mass)^−0.25^ (Stahl 1967), in accordance with McMahon’s theory, ensuring that the metabolic machinery gets adequately supplied with oxygenated blood as animal body size changes. However, the kinetics of various myosin isoforms that constrain heart and respiratory rates scale to body mass with more modest negative exponents, the MyHC kinetic changes being too small in small animals and too big in large animals. This is compensated in the ventricle by the expression of the fast α-cardiac MyHC in small eutherians (Lompre et al. 1981; Kessler-Icekson et al. 1988) and small marsupials (Hoh et al. 2007a) but β-slow MyHC in large animals. In laryngeal muscles, this is compensated in animals at the lower end of the size spectrum by recruiting faster MyHCs not expressed in their limb muscles: 2B MyHC in cats (Rhee and Hoh 2008) and dogs (Wu et al. 2000b) and EO MyHC in rats (Rhee et al. 2004) while in large animals like horses (Rhee et al. 2009) and baboons (Rhee and Hoh 2008) laryngeal muscle speed is reduced by expressing predominantly β-slow and 2A MyHCs. These variations in MyHC expression across species illustrate the phylogenetic plasticity of muscle, i.e. their capacity to adapt to changing functional demands during phylogeny.

The functional demands on different types of muscle across the species varies considerably. Somitic muscles must deal with the postural, locomotory and energetic demands of living in a gravitational environment. The four types of fibres offer a wide range of speeds and fatiguability to satisfy these needs. They consume a very large proportion of the organism’s total energy expenditure, so that minimization of energy expenditure is very important. Hence, the deployment of slow fibres of low tension cost in postural muscles and the availability of three fast MyHCs with a wide range of speed, power and fatiguability for locomotion at different speeds and other motor needs. The physiological plasticity of fibre types allows them to adapt to the pattern of use and to hormonal changes. Energy expenditure during locomotion is minimized by elastic energy saving (Biewener 1998; Alexander 2002), most likely by deploying 2x fibres, as suggested above, being implemented in large eutherian mammals by global elimination of 2b fibres via phylogenetic plasticity of muscle and by selective loss of 2b fibres in muscles involved in elastic energy saving in hopping marsupials.

The effects of functional demand on MyHC expression are more complex in craniofacial muscles. For EOMs, the functional demands are extremely complex, requiring a panel of twelve MyHCs (Hoh 2021). However, functional demands on EOMs are very similar in nature across the species, there is little variation in the repertoire of MyHC express in EOMs of different animals. Airway protection in small animals demands very fast laryngeal muscles, which is met by acquiring EO MyHC expression in laryngeal adductors (Hoh 2010). The functional demands on jaw-closing muscles across different mammalian species are the most complex. Mammals in different ecological niches need to adapt to the different methods of procuring and masticating a wide variety of foods. Mammals inherited the powerful masticatory MyHC from their synapsid ancestors, but many taxa have replaced it with one or more of the cardiac and limb MyHCs with properties more appropriate for the acquisition and mastication of their very diverse types of food. Jaw-closer muscles thus have the most varied pattern of MyHC expression across the species, with a repertoire that includes masticatory MyHC and all skeletal and cardiac MyHC isoforms except EO MyHC (Hoh 2002; Hoh et al. 2006), expressing limb fast and β-slow MyHCs in rodents, masticatory MyHC in carnivores, α-cardiac MyHC in macropods, β-slow MyHC in ruminants, and a complex mixture of MyHCs in rabbits and humans (Hoh 2002), a remarkable instance of phylogenetic plasticity of muscle.

### Phylogenetic perspective on the mechanisms regulating MyHC expression

Of the mechanisms regulating MyHC gene expression in mammals, the roles of myoblast lineage and of thyroid hormone are phylogenetically the most ancient while the role of neural impulse patterns is the most recent. Distinct lineages of progenitor myoblasts giving rise to slow and fast muscle fibres have also been found in zebrafish (Devoto et al. 1996) and birds (Stockdale 1992). Muscles of adult fish (Watabe 2000), amphibian (Lannergren and Hoh 1984; Lutz et al. 1998) and bird (Bandman and Rosser 2000) express several myosin isoforms which differ from those in immature animals (Focant et al. 1992; Nasipak and Kelly 2008; Bandman and Rosser 2000), the developmental transition being mediated by the surge in thyroid hormone which occurs in almost all vertebrates (Hulbert 2000). This occurs in trout during development from parr to juvenile trout (Coughlin et al. 2001), in amphibians during metamorphosis (Chanoine et al. 1990) and in chickens after hatching (Gardahaut et al. 1992). These changes in MyHC gene expression equip the animal with a range of fibre types for an active adult life.

Thyroid regulation of fast MyHC expression in adult mammalian muscles has its origin in ectotherms. Seasonal fluctuations in ambient temperature have led to adaptative changes in fish swimming speeds. Low ambient temperature would reduce their speed, but cold acclimation leads to an increase in fish swimming speed (Johnston et al. 1985, 1990) associated with shifts in MyHC (Watabe 2002) and MLC (Langfeld et al. 1991) isoform expression, compensating for the reduced motility at the lower temperature (Cole and Johnston 2001; Watabe 2002; Tao et al. 2004; Little et al. 2013; Garcia de la Serrana et al. 2018). These changes are mediated by an increase in thyroid hormone level (Little and Seebacher 2013; Little 2021). A seasonal variation in fibre-type distribution favouring faster fibres in winter occurs in the toad (Hoh et al. 1994). It has been postulated that the thyroid induced response to cold is the steppingstone to the evolution of endothermy (Little and Seebacher 2014a).

In sharp contrast to the thyroid regulation of skeletal muscle MyHCs during early vertebrate phylogeny, there is no evidence for thyroid regulation of cardiac MyHC expression in amphibians (Gauthier et al. 2000; Garriock et al. 2005) and birds (Pauletto et al. 1986) in response to the thyroid induced enhanced metabolism. The Xenopus ventricular α-MyHC gene, unlike its mammalian orthologue, does not have a TRE in its promoter (Garriock et al. 2005). Thyroid regulation of cardiac MyHCs is therefore an innovation unique to mammals. However, increased demand for cardiac output in cold-acclimated fish is met by a higher maximum heart rate facilitated by a higher sarco-endoplasmic reticulum Ca^2+^-ATPase (SERCA) activity (Little and Seebacher 2014b) without enhancing the intrinsic contractility of the myocardium.

The capacity of motor nerves in regulating muscle MyHC gene expression in mature animals is uniquely mammalian. Neural regulation confers physiological plasticity to muscle fibre types in mammals, enabling their muscles to adapt to the pattern of use during the lifetime of the animal rather than through intergenerational change via phylogenetic plasticity. Neural influence does play some role in regulating MyHC expression in developing but not in mature avian muscles. During avian development, neonatal myosin is first replaced by adult myosin at the endplate zone, the change progressively moves towards the fibre ends, the reverse occurring following denervation, suggesting that trophic factors emanating from the motor endplate may be involved in myosin regulation (Rosser et al. 2000). Innervation is essential for the normal expression of avian slow MyHC2 during the development of avian slow-tonic muscle (DiMario and Stockdale 1997). This is mediated by neural impulse activity via the NFAT pathway (Crew et al. 2010). Stimulating a developing slow-tonic muscle with a fast neural impulse pattern can partially induce the expression of fast muscle characteristics (Fournier le Ray et al. 1989). Limited plasticity has been observed in cross-innervated avian hatchlings: reinnervation of a slow muscle by a fast nerve partially transformations its myosin ATPase activity towards that of the fast muscle (Syrový and Zelená 1975) and the reverse occurs when a fast muscle in a hatchling is reinnervated by a slow nerve (Hník et al. 1977). Cross-innervation of regenerating fast and slow-tonic avian muscles also leads to the transformation of their myosin ATPase histochemical properties (Kikuchi et al. 1986). However, cross-innervation experiments on adult avian (Feng et al. 1965; Hník et al. 1967) and amphibian (Close and Hoh 1968a) muscles shows that their fast-twitch and slow-tonic muscle fibres are refractory to neural regulation.

The ability of androgens to influence MyHC expression is found in laryngeal muscles of the Xenopus laevis, which are sexually dimorphic: all male laryngeal muscle fibres express a laryngeal specific MyHC while female laryngeal muscle expresses predominantly slow MyHC. Androgen exposure stimulates laryngeal specific MyHC in female Xenopus laryngeal muscles (Catz et al. 1995). Androgens are also involved in controlling the contractile properties of muscles belonging to a different allotype, the forelimb muscles of male amphibians involved in amplexus, though the likely basis that these properties are due to MyHC changes has yet to be determined (Kampe and Peters 2013). In mammals this androgen sensitivity of MyHC expression is found in muscles of yet another allotype, the jaw muscles of rodents (Lyons et al. 1986; Eason et al. 2000; Reader et al. 2001).

### Future directions

Although the pattern of MyHC expression in muscles of different allotypes and the regulation of MyHCs by neural and hormonal mechanisms are established in outline, a considerable amount of work has yet to be done in the future to enhance our knowledge of the molecular mechanisms in this area. Suggestions for future work on specific topics of interest have been made passim in this review. The network of transcription factors in fibres of various ontotypes that determine their different adaptive ranges in MyHC expression needs to be determined. Craniofacial muscles show considerable species variations in MyHC gene expression. Much work also needs to be done to elucidate the transcription factors and cis-regulatory regions of the various MyHC genes in different species that underlie their expression in muscles of different allotypes. It would also be interesting to compare the molecular mechanisms of MyHC regulation by androgens in muscles of different allotypes.
